# Ductile Fracture Prediction in Cross-Wedge Rolling of Rail Axles

**DOI:** 10.3390/ma14216638

**Published:** 2021-11-04

**Authors:** Tomasz Bulzak

**Affiliations:** Mechanical Faculty, Lublin University of Technology, 36 Nadbystrzycka Str., 20-618 Lublin, Poland; t.bulzak@pollub.pl; Tel.: +48-81-538-4246

**Keywords:** rail axles, cross-wedge rolling, cracking

## Abstract

In the process of cross-wedge rolling, axial-symmetric forgings are formed using wedge tools. These tools may be flat- or roll-shaped. This article presents two methods of cross-wedge rolling of rail axles, traditional and multi-wedge, as well as their advantages and disadvantages. Two cross-wedge rolling processes are modelled numerically using Simufact Forming. Numerical results are then verified by experiments performed on a flat wedge rolling mill. Results obtained with the two rolling methods are compared in terms of material fracture, force parameters, effective strain and thermal conditions during rolling. Results show that material fracture poses a serious problem in these rolling processes. It is found that the Cockcroft–Latham ductile fracture criterion does not predict material fracture correctly. Results demonstrate that the fracture of railway axles in cross-wedge rolling can be best predicted by the fracture criteria developed by Ayada, Brozzo, Ko, Rice and Tracey.

## 1. Introduction

Rail axle is one of the basic elements of a wheel set of a rail vehicle. A report by Persistence Market Research prognosticates that the compound annual growth rate CAGR for the market of rail axles and wheels ought to be 5.6% by 2026. Should the growth remain at the assumed level it would reach the value of USD 5533.8 million [1]. The growth of the market of rail axles and wheels results from the development of high-speed rail systems, as well as the aim of limiting exhaust emissions generated by automobiles. Currently, rail axles of similar quality are offered by numerous producers, which leads to a lowering of the production costs by introducing more efficient and less energy-consuming technologies.

In the process of manufacturing rail axles, the technologies of forging and, to a smaller extent, rolling are used [2]. In the case of forging two types ought to be mentioned: open die forging on a hydraulic press and forging in swaging machines [3,4]. The disadvantage of both of those processes is a low effectiveness of forging compared to rolling. Rolling-based technologies allow one to obtain a greater efficiency than in the case of forging. In the process of rail axles production, the most favourable methods are computerized numerical control (CNC) skew rolling and cross-wedge rolling on flat- or roll-shaped tools. CNC skew rolling technology seems to be of great potential; a CNC skew rolling mill was built at Lublin University of Technology (Poland) in order to develop this technology [5].

The development possibilities in the case of cross-wedge rolling are much greater due to the fact that several producers offer cross-wedge rolling mills. The limitation to this process is the size of the tools necessary. In the case of flat tools, their length must exceed 6 m, whereas in the case of roll-shaped tools their diameter ought to be about 2 m. No currently available rolling mill allows one to use tools of that size. This problem can be solved by technology that enables using shorter tools, as provided by the multi-cross-wedge rolling process.

The cross-wedge rolling process for producing railway axles was investigated by the finite element method (FEM) [6]. In this study, the authors focused on the formation of internal cracks in cross-wedge-rolled parts. Railway axles are key elements in the design of rolling stock. Many authors have independently shown that during operation, railway axles are subjected to variable loads that may cause damage [7,8,9]. The main problem in the production of railway axles by cross-wedge rolling is the formation of cracks along the axis of a rolled part. Railway axle defects such as micro cracks formed in cross-wedge rolling may lead to a faster growth of fatigue cracks. Therefore, the assessment of crack formation probability during the production of axles by cross-wedge rolling seems to be crucial for rail transport safety.

The objective of this study is to investigate whether railway axles can be produced by two cross-wedge rolling methods. Another objective is to assess the possibility of predicting undesired material fracture with the use of popular ductile fracture criteria.

## 2. Materials and Methods

**Figure 1:**
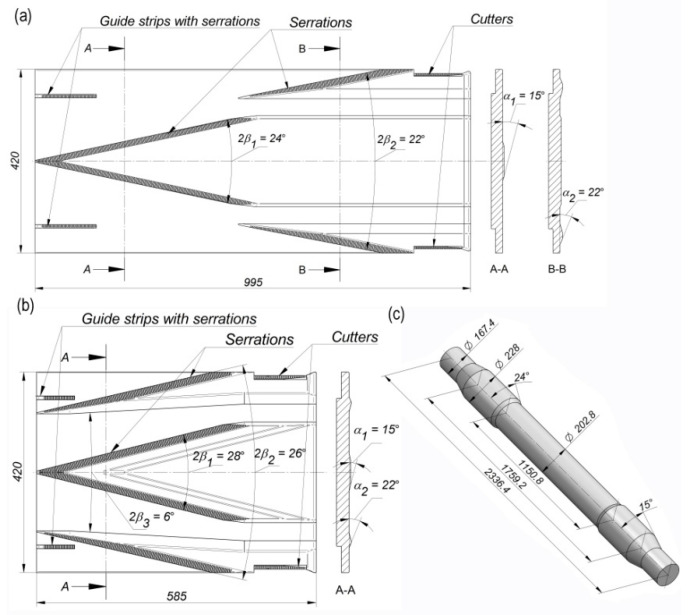
Scheme of instrumentation and the rail axle: (**a**) 1:6 scale model of traditional wedge tool; (**b**) 1:6 scale model of multi-wedge tool; (**c**) 1:1 scale model of rail axle.

This article presents a comparison of cross-wedge rolling of rail axles using the traditional and multi-wedge tools. The 1:6 scale model tools for the traditional method is shown in Figure 1a, whereas the 1:6 scale model of the tools for the multi-wedge method is shown in Figure 1b. The shape and dimensions of the 1:1 scale model of a rail axle are shown in Figure 1c. The 1:6 scale resulted from the size of the rolling mill and furnace available.

In the case of the traditional tool, first the 202.8 mm diameter middle step of the rail axle is formed, then the 167.4 mm diameter end steps. In the case of cross-wedge rolling with multi-wedge tools, the end steps and the middle step are rolled simultaneously. Multi-wedge rolling allows one to shorten the tool by 41%. A significant parameter of the multi-wedge tool is the *β*_3_ angle value. The *β*_3_ angle is the inner flare angle of the outer wedges forming the end steps of the axle. Proper selection of the angle *β*_3_ compensates for the elongation of the middle step of the forging during the rolling process. Should the value of the *β*_3_ angle be too small, the forging may be seized between the end wedges.

**Figure 2:**
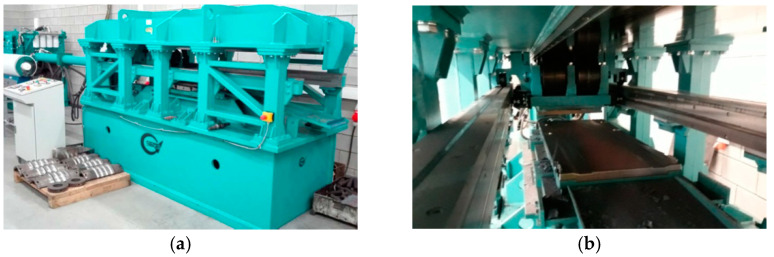
Test stand: (**a**) flat-wedge rolling mill; (**b**) work space with a tool.

Technologies of cross-wedge rolling using two tool sets (Figure 1) were realised on a flat-wedge laboratory rolling mill (Lublin University of Technology, Lublin, Poland) (Figure 2). In the case of both technologies, the billet material was ∅38 × 300 mm 42CrMo4 grade rods. The rod billet was preheated to 1150 °C. The wedge tool temperature was 100 °C and was constant throughout the simulation. The rolling process was also modelled using the finite element method (FEM) in Simufact Forming. In the numerical modelling, the tool models presented in Figure 1 were utilized. In both experiment and modelling, one wedge moved at the constant speed of 300 mm/s. A constant friction model, with the friction factor equal to 0.9, was used to describe the contact conditions in the FEM environment. Thermal parameters were described using the heat transfer coefficient between the tools and the billet, equal to 20 kW/m^2^K. The heat transfer coefficient between the billet and the environment was described with the coefficient 50 W/m^2^K. The material model of 42CrMo4 steel is described by the following equation:

(1) $$\sigma_{p}=4628.83\;e^{-0.0034T\;}\varepsilon^{\left(-0.00000509T-0.036\right)}e^{\left(-0.0000046T-0.019/\varepsilon \right)}{\dot{\varepsilon }}^{\left(0.00018T-0.046\right)}$$

where: *σ_p_*—yield stress, MPa; *T*—temperature, °C; *ε*—effective strain, $\dot{\varepsilon }$—strain rate, s^−1^; *e*—Euler’s number.

The fracture of railway axles in cross wedge rolling was FEM-predicted based on the empirical normalised Cockcroft–Latham ductile fracture criterion, which is expressed as:

(2) $$\;f_{nCL}=\int_{0}^{\varepsilon }\frac{\sigma_{1}}{\sigma_{i}}d\varepsilon \geq f_{c}$$

where: *f*—ductile fracture criterion value, *ε*—effective strain, *σ*_1_—maximum principal stress, *σ_i_*—effective stress, *f_c_*—limit value of the ductile fracture criterion. 

**Figure 3:**
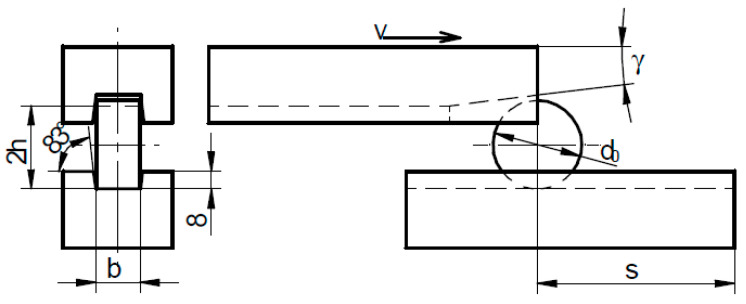
Principle of rotary compression test [10].

According to the Cockcroft–Latham criterion, the material fracture occurs when the fracture criterion value *f* is greater than or equal to the limit value of this criterion, *f_c_*. The limit values *f_c_* for the 42CrMo4 steel were calculated via a rotary compression test, the principle of which is shown in Figure 3. To calculate the limit fracture criterion value *f_c_*, it is necessary to experimentally determine the number of revolutions *n* or the stroke *s* at which fractures occur in real conditions. The calculated value corresponds to the limit criterion value at which material fracture occurs. The numerical modelling of the rotary compression test and its experimental verification were carried out under the same conditions as the cross-wedge rolling process for railway axles. The rotary compression test was performed using discs with the initial dimensions of ∅40 × 20 mm; after the test, the discs had a height of 2*h* = 38 mm.

## 3. Results and Discussion

**Figure 4:**
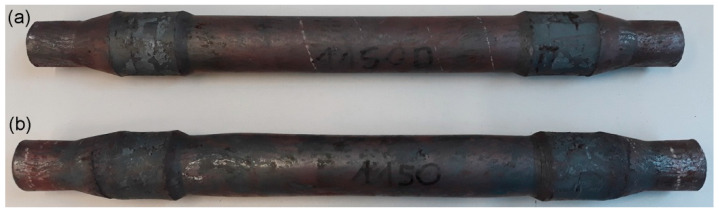
Rail axles rolled: (**a**) with traditional tools; (**b**) with multi-wedge tools.

Figure 4 presents rail axles (Lublin University of Technology, Lublin, Poland) obtained in laboratory testing using the tool set presented in Figure 1. The shape of the axle rolled using traditional tools is better than in the case of multi-wedge tools. The results of measurements made on individual sections of produced railway axles are listed in Table 1. In the case on multi-wedge tools, the middle step of the rolled axle has two visible protrusions. The diameter of the axle rolled with the traditional method is constant throughout the entire length of the middle step. The fact that the diameters d3 and d5 are larger in the axle rolled with the use of multi-wedge tools results from the material moving backwards to the centre due to its reduction between the outer and the middle wedge tool. In addition to that, the surface of the middle wedge tool was lowered in order to reduce the susceptibility of material to cracking. The lowered wedge tool surface led to the creation of open space for material flow, which resulted in the formation of thickened areas in the diameters d3 and d5.

**Figure 5:**
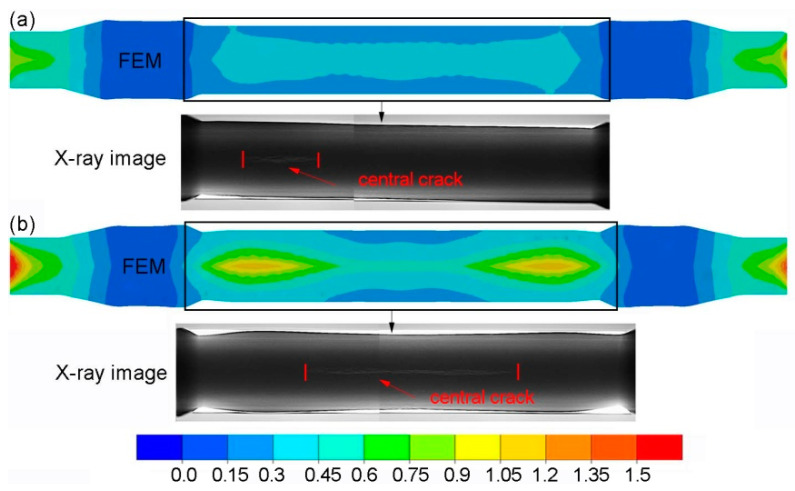
Distribution of the Cockcroft–Latham damage criterion and X-ray image of the rolled axle: (**a**) with traditional tools; (**b**) with multi-wedge tools.

The obtained axles were examined for the occurrence of inner cracks using an X-ray method. Figure 5 presents the X-ray images of the rail axle forgings as well as the distributions of the Cockcroft—Latham damage criterion obtained in the numerical modelling. The X-ray images comprised two photographs due to the maximum size of the object photographed. The distributions of the damage criterion obtained from FEM indicate that a greater risk of cracking occurs during rolling with multi-wedge tools. The maximum value of the damage criterion in the middle step of the axle reached 0.6 during rolling with traditional tools. In the case of the axle rolled with multi-wedge tools, in two areas located at the ends of the middle step, the value of the damage criterion increased to 1.2. The limit value of the Cockcroft¬–Latham damage criterion for the 42CrMo4 grade steel was determined in a rotational compression test. The results of the rotary compression test conducted with different temperatures are given in Table 2. The results indicate that the limit value of the Cockcroft–Latham criterion, after which material cohesion loss will occur for the 42CrMo4 grade steel at 1150 °C is 2.68. The results of the rotational compression test and FEM indicated that cracking would not occur in axles rolled with both tool sets. The X-ray image of the central part shows that regardless of the value of the fracture criterion, cracking occurred in axles rolled with both tool sets. Moreover, the localization of the cracking in both cases was not compliant with the damage criterion distribution obtained using FEM. The results demonstrate that the Cockcroft–Latham ductile fracture criterion does not predict the location of fracture initiation correctly.

**Figure 6:**
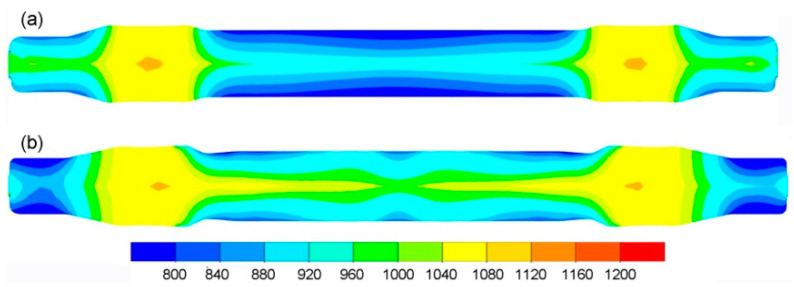
Temperature distribution in °C of the rolled axle: (**a**) with traditional tools; (**b**) with multi-wedge tools.

The data in Table 2 demonstrate that the limit value of the normalised Cockcroft–Latham criterion, *f_cnCL_*, depends on the temperature. Consequently, Figure 6 shows the distribution of temperature in the produced railway axles. It can be observed that the highest decrease in material temperature occurs at the surface of the produced axles. The lowest temperature drop occurs in the largest diameter regions of the axle, due to their limited contact with the tools. As far as the conventional rolling method is concerned, the highest drop in temperature is located in the central part of the axle. This stems from the fact that the workpiece centre was in contact with much colder tools throughout the entire duration of the rolling process. As for the multi-wedge tool rolling method, the highest drop in temperature can be observed on the workpiece ends. These regions of the workpiece were subjected to deformation from the very beginning of the rolling process, and they had the longest contact with the much colder tools. Although the central part of the workpiece was also deformed from the start of the rolling process, the lowering of the middle wedge tool led to limited heat transfer to the cooler tools. The lower temperature drop in this rolling case also results from a shorter duration of the rolling process. In the central part of both axles (crack location), the temperature after rolling ranges 920–960 °C for the conventional method and 960–1040 °C for the multi-wedge tool method. Considering the limit Cockcroft–Latham criterion values *f_cnCL_* for these temperature ranges, no cracks should occur in these regions.

The above results demonstrate that the normalised Cockcroft–Latham criterion, which is the default used in FEM software for fracture prediction, failed to predict material fracture in the cross-wedge rolling process for railway axles. Therefore, the suitability of other criteria for fracture prediction in forming processes was assessed. Table 3 presents the mathematical equations describing the analysed phenomenological ductile fracture criteria.

**Table 3:** Selected criteria of ductile fracture used for the presented analysis [10].

Abbreviation	Criterion	Formula	No
FREU	Freudenthal (1950)	$f_{FREU}=\int_{0}^{\varepsilon }\sigma_{i}d\varepsilon$	(3)
CL	Cockroft and Latham (1968)	$f_{CL}=\int_{0}^{\varepsilon }\sigma_{1}d\varepsilon$	(4)
RT	Rice and Tracey (1969)	$f_{RT}=\int_{0}^{\varepsilon }\exp\left(\frac{3}{2}\eta \right)d\varepsilon$	(5)
BROZ	Brozzo et al. (1972)	$f_{BROZ}=\int_{0}^{\varepsilon }\frac{2\sigma_{1}}{3\left(\sigma_{i}-\sigma_{m}\right)}d\varepsilon$	(6)
OYAN	Oyane (1972)	$f_{OYAN}=\int_{0}^{\varepsilon }\left(1+A\eta \right)d\varepsilon$	(7)
ARGO	Argon et al. (1975)	$f_{ARGO}=\int_{0}^{\varepsilon }\left(\sigma_{m}+\sigma_{i}\right)d\varepsilon$	(8)
KO	Ko et al. (2007)	$f_{KO}=\int_{0}^{\varepsilon }\frac{\sigma_{1}}{\sigma_{i}}\langle 1+\eta \rangle d\varepsilon$	(9)
AYAD	Ayada (1984)	$f_{AYAD}=\int_{0}^{\varepsilon }\eta d\varepsilon$	(10)
ZHAN	Zhan et al. (2009)	$f_{ZHAN}=\int_{0}^{\varepsilon }\left(\sigma_{i}-\sigma_{m}\right)d\varepsilon$	(11)

Where: A—material constant (after [11] A = 0.424), *σ_m_*—mean stress, *σ_i_*—equivalent stress, *σ_1_*—maximal principal stress, *η*—stress triaxiality; 〈〉 is the Macaulay bracket.

**Figure 7:**
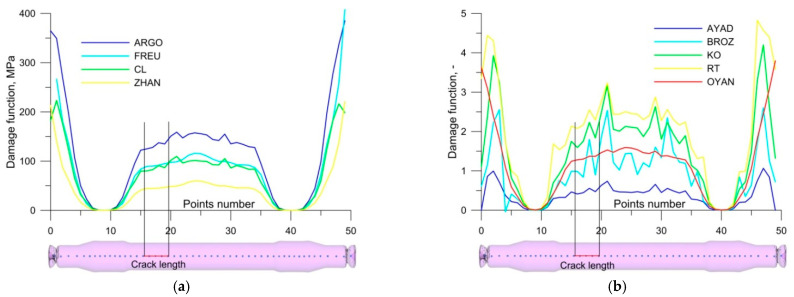
Damage function values for railway axles rolled with traditional tools: (**a**) dimensional criteria (MPa); (**b**) dimensionless criteria.

Figure 7 shows the changes in the values of the ductile fracture criteria along an axle rolled with the use of conventional tools. Regarding the dimensional (MPa) criteria, none of the criteria from Argon [12], Freudenthal [13], Cockcroft and Latham [14] and Zhan [15] (Figure 7a) identify the crack location correctly. The dimensionless criteria (Figure 7b) identify the location of the fracture with greater accuracy. The Oyane criterion [16] is the only one to reach the highest value in the centre of the axle. The other dimensionless criteria exhibit increased values at some distance from the centre of the axle, where the material fracture did actually occur. All the analysed criteria have very high values at the axle ends. This phenomenon results from the impact of cutters that cut off end waste during the rolling process.

**Figure 8:**
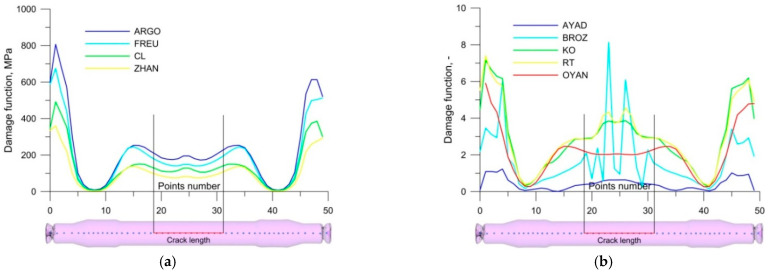
Damage function values for railway axles rolled with multi-wedge tools: (**a**) dimensional criteria (MPa); (**b**) dimensionless criteria.

Figure 8 shows the changes in the values of the ductile fracture criteria along a railway axle rolled with the use of multi-wedge tools. None of the dimensional criteria (MPa) (Figure 8a) predicts the fracture location correctly. The dimensionless criteria (Figure 8b) predict the fracture location more accurately. As in the previous case, the Oyane criterion fails to identify the fracture location correctly. The results of the rotary compression test for the fracture criteria (Ayada [17], Brozzo [18], Ko [19], Rice and Tracey [20]) that managed to identify fracture location correctly were then used to determine the limit values of these criteria. By knowing the limit values of these criteria, it was possible not only to identify fracture location, but also to assess the probability of fracture formation in this region. Limit values of the ductile fracture criteria by Ayada, Brozzo, Ko, Rice and Tracey are listed in Table 4. In the numerical simulations the crack was located on both sides of the axis symmetrically (rolling with traditional tools). In the experimental conditions it is difficult to obtain ideal billet alignment conditions such as in the numerical simulation. As a result, skewing of the billet may occur during rolling. In the case of rolling with traditional tools this is very likely to happen as the material is rolled from the centre (the material is gripped by the tools at one point) and, in addition, it is relatively long, which makes it easier to bend the material. In the case of multi-rolling tools, the risk of skewing of the billet at the initial stage of rolling is not so great as the material is gripped at the same time at three points, which significantly reduces the skewing of the billet.

Table 5 gives the maximum values of the ductile fracture criteria by Ayada, Brozzo, Ko, Rice and Tracey, as calculated in material fracture loci. Moreover, Table 5 gives the limit values of the damage function for the temperatures toward the end of the rolling process. The data in Table 5 demonstrate that the analysed criteria have considerably exceeded their limit values for the temperatures toward the end of the rolling process. The limit damage function values for the temperature at the beginning of the rolling process are, in most cases, exceeded or similar to the limit values for this temperature. Summing up, the ductile fracture criteria developed by Ayada, Brozzo, Ko, Rice and Tracey can be used to predict fracture in the cross-wedge rolling process for railway axes.

Of the four criteria that correctly predicted the location of the crack, in three of them, the sub-integral function was based on the stress triaxiality. The Oyane criterion, also based on the stress triaxiality, did not indicate the correct crack location. The reason for this may be the fact that this criterion uses a correction factor A. According to the literature [19], this factor is assumed to have a value of A = 0.424. In [21], the authors state that this factor is used to a reasonable extent to obtain a better correlation between experimental and numerical tests. Therefore, it can be assumed that the Oyane criterion can be applied to different forming processes only after the coefficient A has been correctly determined. The Brozzo criterion also indicated the correct crack location despite the fact that it is not based on the stress triaxiality. The Brozzo criterion, like the other criteria, is also based on the value of the equivalent stress and the mean stress. A common element of fracture criteria correctly indicating the location of a crack is the use of a stress state index relating to the equivalent stress.

**Figure 9:**
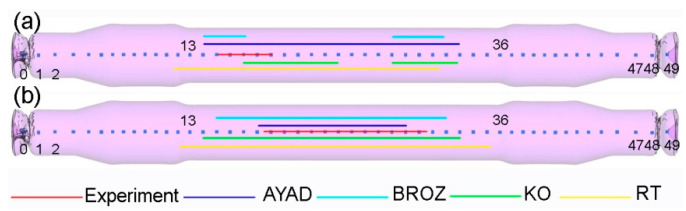
Crack lengths determined based on the four ductile fracture criteria and the limit value of these criteria obtained in the rotary compression test: (**a**) traditional tools; (**b**) multi-wedge tools.

Figure 9 shows the crack lengths determined based on the four ductile fracture criteria and the limit value of these criteria obtained in the rotary compression test. In the case of rolling with traditional tools, the crack length was best estimated based on the Brozzo criterion. In the case of the multi-wedge method, the crack length was best estimated based on the Ayad criterion.

**Figure 10:**
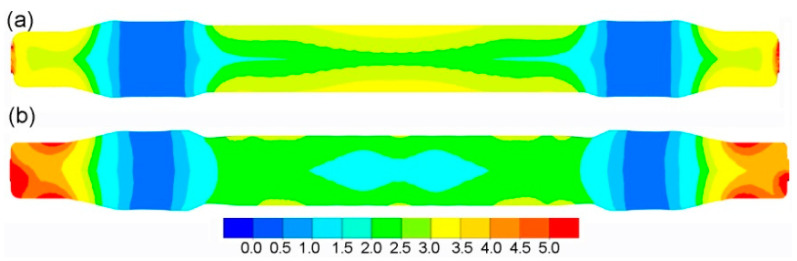
Distribution of effective strains in a railway axle rolled with: (**a**) traditional tools; (**b**) multi-wedge tools.

Figure 10 shows the distribution of effective strains in produced axles. As a result of using different wedge tools, the strain distributions and values differ. In the central part of the axle, the strains are higher when the rolling process is performed with conventional tools. The strains on the axle ends are higher for the rolling process performed with the use of multi-wedge tools. In the largest diameter region of the axle (wheel seat), the strains are the lowest and have similar values for both rolling methods. The strains are more uniform in the centre of the axle for the rolling process performed with the use of multi-wedge tools. On axle ends, a greater strain uniformity is achieved when the rolling process is performed with conventional tools.

**Figure 11:**
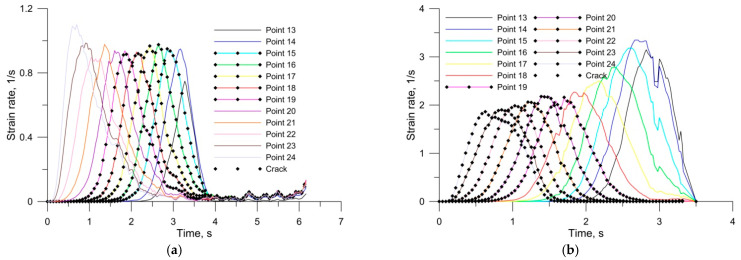
Distribution of effective strain rates in a railway axle rolled with: (**a**) traditional tools; (**b**) multi-wedge tools.

Figure 11 shows the distribution of strain rate in the points located in the middle step axis of the rolled axles (Figure 9). In the case of traditional tool rolling (Figure 11a), the strain rate in the middle step axis along the whole length takes the value of 1 s^−1^. In this case, the value of the strain rate in places where cracking occurred (points 15–19) and in places where there was no crack (points 13, 14, 20–24) is at a similar level. In the case of rolling with traditional tools, no relationship was observed between the value of strain rate and crack location. In the case of rolling with multi-wedge tools (Figure 11b), the strain rate in the middle step is not uniform. The centre of the middle step is formed at strain rates of the order of 2 s^−1^, while the ends of the middle step are formed at strain rates of the order of 3 s^−1^. In this rolling case, the area where cracking occurred was rolled at lower strain rates (points 19–24). In the multi-wedge method, material cracking occurred at higher strain rates than in the traditional method. The results presented did not confirm a clear relationship between material cracking and strain rate values.

**Figure 12:**
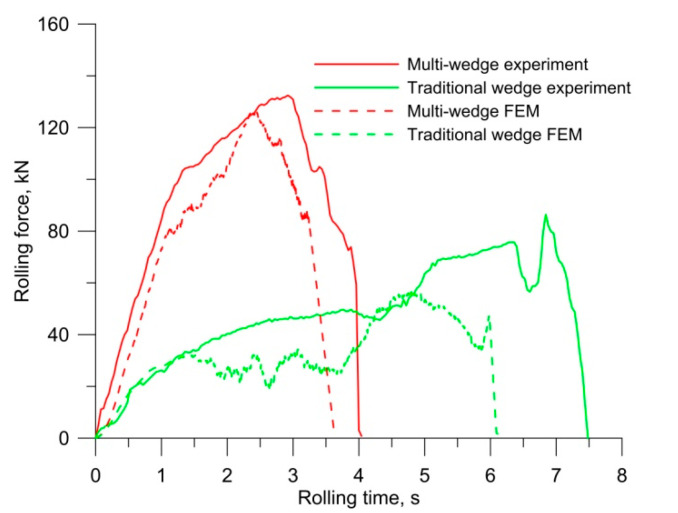
Comparison of rolling forces with different tools.

Figure 12 presents the progression of rolling forces registered during experimental and FEM testing. The schemes indicate that that the process of rolling with multi-wedge tools is quicker (shorter rolling time). The maximum value of the rolling force using multi-wedge tools equals 132 kN and exceeds the maximum rolling force with traditional tools (86 kN) by 53%. Different rolling paths for both methods result in the energy performed by both sets of tools being comparable. The energy for the traditional tools equals 89.7 kJ, whereas for multi-wedge tools it is 94.5 kJ. In the rolling process performed with multi-wedge tools, very high rolling forces cause undesired elastic strains of the rolling mill, which, in turn, leads to changes in the distance between the tools. As a result, the diameters of axes rolled with the use of multi-wedge tools may be larger than those assumed at the process design stage. This hypothesis is confirmed by the results of diameters of the produced axles given in Table 1. These data clearly show that the axle rolled with the multi-wedge tool method has larger diameters of individual regions.

The agreement between the FEM and experimental results is higher for the rolling process performed with multi-wedge tools. The determination coefficient R^2^ describing the agreement between the numerical model and the actual data is 0.87 for the multi-wedge tool method and 0.70 for the conventional method. High qualitative agreement between FEM and experimental forces can be observed for both analysed rolling cases. The numerical and experimental forces in the multi-wedge tool method show a high agreement. This results from the fact that, despite the material fracture in the experiment, the ovalization of the cross section does not lead to increased rolling resistance in spite of the lowering of the middle wedge tool surface. As for the conventional rolling method, the forces in the experiment are much higher than in the FEM analysis. This is due to material fracture in the centre of the axle, which led to the ovalization of the cross section in the fracture locus. The cross-sectional ovalization resulted in increased rolling resistance, which, in turn, led to an increase in rolling force.

## 4. Conclusions

This paper presented two methods of cross-wedge rolling of rail axles. The obtained results indicate that a serious problem in the process of rolling rail axles is material fracture. To solve the problem of material fracture in the cross wedge rolling of railway axles, it is necessary to investigate the effect of rolling temperature and tool design modification. Rolling with traditional and long tools is problematic due to the required size of the rolling mill and tools. The method of rolling with multi-wedge tools appears to be the perfect solution. The results of this study lead to the following conclusions:

- There is a very high risk of material fracture in the cross-wedge rolling process for railway axles;
- Railway axles rolled with the use of conventional tools exhibit higher dimensional accuracy than the axles produced with multi-wedge tools;
- The Cockcroft–Latham ductile fracture criterion, which is the default used in FEM software, does not predict material fracture correctly;
- The dimensionless criteria developed by Ayada, Brozzo, Ko, Rice and Tracey can be employed to detect the location of cracks in railway axles produced by cross-wedge rolling;
- The rotary compression test and the ductile fracture criteria by Ayada, Brozzo, Ko, Rice and Tracey make it possible to determine fracture initiation in the cross-wedge rolling process for railway axles;
- Due to a shorter process duration, the multi-wedge tool rolling method ensures a reduced material cooling during rolling;
- Effective strains in individual sections of the railway axle depend on the rolling method applied;
- Higher rolling forces must be applied in the multi-wedge tool rolling method;
- In both analysed cross-wedge rolling methods, the rolling energy is similar.

In order to implement the cross-wedge rolling process in the industry it is necessary to modify the tools and select proper technological parameters to eliminate cracks and enhance the shape and dimensions of the axles.

## Figures and Tables

**Table 1:** Dimensions of produced railway axles in a scale of 1:6.

Dimension	d1 (mm)	d2 (mm)	d3 (mm)	d4 (mm)	d5 (mm)	d6 (mm)	d7 (mm)	L1 (mm)	L2 (mm)	L3 (mm)
CAD	27.9	38	33.8	33.8	33.8	38	27.9	182.4	293.2	389.4
Traditional tools	28.3 ± 0.29	38.0 ± 0.08	34.3 ± 0.30	34.3 ± 0.06	34.6 ± 0.26	38.0 ± 0.05	28.3 ± 0.18	181.2 ± 0.65	291.6 ± 0.96	387.8 ± 0.91
Multi-wedge tools	28.7 ± 0.50	38.3 ± 0.04	35.6 ± 1.15	34.6 ± 0.56	35.3 ± 1.66	38.3 ± 0.14	28.9 ± 0.53	183.1 ± 0.53	292.4 ± 0.87	388.4 ± 0.69
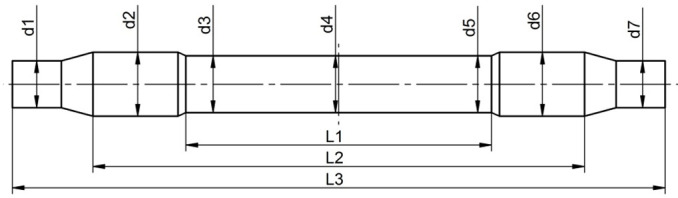										

**Table 2:** Limit value of normalised ductile fracture criterion according to Cockcroft–Latham determined in the rotary compression test.

Temperature T (°C)	900	950	1000	1050	1100	1150	1200	*fc = f(T)*	*R* ^2^
*f_cnCL_*	1.15	1.28	1.55	1.85	2.2	2.68	3.56	*f_cnCL_* = 0.0379*e*^0.0037*T*^	0.98

**Table 4:** Limit value of damage function according to Ayada, Brozzo, Ko, Rice and Tracey determined in the rotary compression test.

Temperature T (°C)	900	950	1000	1050	1100	1150	1200	*fc = f(T)*	*R* ^2^
*f_cAYAD_*	0.35	0.39	0.40	0.50	0.56	0.64	0.72	*f_cAYAD_* = 0.0359*e*^0.0025*T*^	0.98
*f_cBROZ_*	1.02	1.13	1.15	1.43	1.64	1.82	2.06	*f_cBROZ_* = 0.1095*e*^0.0024*T*^	0.97
*f_cKO_*	1.95	2.08	2.27	2.70	3.30	3.50	3.98	*f_cKO_* = 0.19*e*^0.0025*T*^	0.97
*f_cRT_*	1.61	1.83	1.89	2.42	2.80	3.21	3.69	*f_cRT_* = 0.1186*e*^0.0029*T*^	0.98

**Table 5:** Comparison of damage function values and their limit values for temperatures toward the end of the rolling process.

Criterion	Traditional Tools			Multi-Wedge Tools		
	*f*	*fc*	prediction	*f*	*fc*	prediction
AYAD	0.73	0.35	correct	0.63	0.39	correct
BROZ	2.53	1.02	correct	8.14	1.13	correct
KO	3.18	1.95	correct	3.77	2.08	correct
RT	3.23	1.61	correct	4.56	1.83	correct

## Data Availability

Data is contained within the article.
